# Determinants of In-Hospital Mortality Among Type 2 Diabetes Mellitus-Related Admissions in a Tertiary Teaching Hospital

**DOI:** 10.3390/healthcare14030347

**Published:** 2026-01-30

**Authors:** Norfarhana Samsudin, Roszita Ibrahim, Azimatun Noor Aizuddin, Siti Noorain Hamid

**Affiliations:** 1 Department of Public Health Medicine, Faculty of Medicine, Universiti Kebangsaan Malaysia, Kuala Lumpur 56000, Malaysia; norfarhana@hctm.ukm.edu.my (N.S.); azimatunnoor@hctm.ukm.edu.my (A.N.A.); 2 Health Informatics Centre, Hospital Canselor Tuanku Muhriz, Universiti Kebangsaan Malaysia, Kuala Lumpur 56000, Malaysia; 3 Secretariat of Research & Innovation, Faculty of Medicine, Universiti Kebangsaan Malaysia, Kuala Lumpur 56000, Malaysia; sitinoorain@ukm.edu.my

**Keywords:** casemix system, infection, inpatient, mortality determinants, type 2 diabetes mellitus

## Abstract

**Highlights:**

**What are the main findings?**

- In-hospital mortality among T2DM-related admissions was 4.2%, with higher risk observed among older patients (mean age 67.72 years in deceased vs. 65.11 years in survivors).
- Key determinants of mortality included infections and parasitic diseases, respiratory, hepatobiliary/pancreatic, and central nervous system conditions, as well as higher disease severity and comorbidity burden.

**What are the implications of the main findings?**

- Clinical Implication: The strong association between infection-related, respiratory, hepatobiliary, and CNS conditions with mortality among T2DM-related admissions suggests the need for early detection and aggressive management of these comorbidities. Hospitals should implement routine screening and rapid intervention protocols for T2DM-related admissions presenting with these high-risk conditions.
- Healthcare System Implication: The findings highlight the importance of a multidisciplinary care approach among endocrinologists, infectious disease specialists, and intensive care teams to improve survival.

**Abstract:**

**Background/Objectives**: Globally, type 2 diabetes mellitus (T2DM) accounts for about 90% of diabetes cases and contributes to hospital admissions and mortality in Malaysia. Identifying the determinants of in-hospital mortality is crucial for improving clinical management and resource allocation. This study aims to determine the clinical and disease-related determinants of in-hospital mortality among T2DM-related admissions in a tertiary teaching hospital. **Methods**: A cross-sectional study at Hospital Canselor Tuanku Muhriz (HCTM) in Kuala Lumpur involving 2838 T2DM-related admissions from the hospital casemix database. Demographic data, complications, disease group, length of stay, and number of diagnoses were analyzed. Logistic regression assessed factors associated with in-hospital mortality among T2DM-related admissions. **Results**: The in-hospital mortality rate among T2DM-related admissions was 4.2%. T2DM-related admissions resulting in in-hospital death involved individuals with a higher mean age (67.72 years, SD 12.06) compared to admissions that did not result in death (65.11 years, SD 11.03). Significant determinants of mortality included infections and parasitic diseases (aOR = 8.042; 95% CI: 2.999, 21.569; *p* < 0.001), respiratory system (aOR = 3.004; 95% CI: 1.192, 7.571; *p* = 0.020), hepatobiliary/pancreatic (aOR = 3.674; 95% CI: 1.143, 11.871; *p* = 0.029), and central nervous system (aOR = 3.484; 95% CI: 1.236, 9.826; *p* =0.018) conditions, and severity level 3 (aOR = 2.994; 95% CI: 1.464, 6.221; *p* = 0.003). Each additional diagnosis increased the mortality risk (aOR = 1.107; 95% CI: 1.032, 1.189; *p* = 0.005). **Conclusions**: Mortality among hospitalized T2DM-related admissions is driven by severe infections, respiratory, hepatobiliary, and neurological conditions, together with overall disease burden. Early identification of high-risk clinical presentations and a timely multidisciplinary approach may reduce preventable deaths among T2DM patients.

## 1. Introduction

Type 2 diabetes mellitus (T2DM) is a non-communicable disease (NCD) of increasing concern globally. T2DM comprises up to 90% of all diabetes cases [1]. The International Diabetes Federation (IDF) reported that among adults aged 20–79 years in the world in 2021, 537 million were living with diabetes. It is projected that the number will increase to 643 million by 2030 and 783 million by 2045 [2]. This epidemic is growing and diminishes life expectancy [3]. Malaysia is also experiencing this problem, with a worrying rise in the prevalence of T2DM. The latest National Health and Morbidity Survey (NHMS) in 2023 reported that the prevalence of diabetes among adults aged 18 years was 15.6% [4], while 99.5% of patients registered with National Diabetes Registry (NDR) had T2DM [5].

T2DM is characterized by insulin resistance and relative insulin deficiency, leading to a prolonged state of hyperglycemia in the body. This results in various complications, both microvascular and macrovascular. In the long run, T2DM patients are at increased risk of morbidity and mortality [6]. Concurrently, diabetes causes metabolic disturbances that compromise the body’s immune response, as hyperglycemia disrupts the regulation of innate and adaptive immunity, resulting in an impaired ability to combat infections, making T2DM patients susceptible to severe infections [7,8]. These complications often result in prolonged hospitalizations and higher fatality rates in this population despite the primary reason for admission [9,10].

T2DM contributed to nearly 3.4 million deaths globally in 2024. T2DM-related mortality worldwide increased by 142.9% between 1990 and 2019 [11]. Nearer to home, a recent study in Indonesia, which analyzed 610,000 hospitalized T2DM patients, showed an in-hospital mortality rate of 6.6% [12]. In Malaysia, the situation does not differ from global trends, where T2DM patients have been seen to have higher hospitalization rates and increased in-hospital mortality compared to the general population [13]. These findings highlight the need for targeted strategies to identify and manage determinants of mortality among T2DM-related admissions.

In-hospital mortality is one of the key indicators of patient safety and healthcare quality [14,15]. It reflects system-level performance, efficiency of patient care, and quality benchmarking. For specific diseases, it reflects the burden of the disease on the health facility. Recognizing mortality determinants among hospitalized patients with T2DM may assist in creating risk-stratification tools, inform resource distribution, and improve early intervention strategies. Various studies have examined such determinants internationally [16,17], but scarce evidence is found specific to the Malaysian tertiary healthcare context, particularly studies leveraging casemix data to evaluate patient-level and disease-related in-hospital mortality. The present study aims to analyze in-hospital mortality determinants among patients with T2DM admitted to a tertiary teaching hospital in Kuala Lumpur.

## 2. Materials and Methods

### 2.1. Study Design and Setting

This cross-sectional study was conducted at Hospital Canselor Tuanku Muhriz (HCTM), a tertiary teaching hospital located in Cheras, Kuala Lumpur. HCTM is associated with Universiti Kebangsaan Malaysia (UKM) and serves as a major referral hospital within the Klang Valley and surrounding regions. The hospital has a capacity of over 1000 beds and provides comprehensive medical, surgical, and diagnostic services.

This study utilized inpatient data from the casemix database of HCTM, UKM. This study analyzed hospital discharges involving individuals with T2DM, rather than unique patients. Each discharge represents a single hospitalization episode and may include multiple admissions from the same individual. The casemix database classifies patients with similar clinical characteristics and resource utilization into a single homogeneous group. The clinical characteristics include the diagnosis of patient and the procedures undergone by the patient for each episode of care. The important component of the system is morbidity coding, which is a systematic assignment of diagnostic codes using the International Classification of Diseases, 10th Revision (ICD-10), and procedure codes using ICD-9 Clinical Modification (ICD-9-CM) by hospital clinical coders. The codes are then grouped using grouper software, the Malaysian Diagnosis-Related Group (MY-DRG^®^) system, into 22 casemix main groups (CMGs) representing major body systems [18], as demonstrated in Table 1 below. MY-DRG^®^ grouper software provided by Casemix Solutions Sdn. Bhd. (Bandar Kajang, Selangor, Malaysia) was used. Software information is available at https://www.casemix.com.my/, accessed on 20 January 2026. Each CMG, labelled alphabetically from A to Z, corresponds to ICD-10 chapters and reflects organ systems or major clinical categories. Casemix data can be a very valuable source of data to assess in-hospital mortality determinants among T2DM-related admissions. CMG classification was included as a variable to assess its association with mortality among T2DM-related admissions.

### 2.2. Study Population

The study population comprised all hospital discharges involving individuals aged 18 years and above from HCTM between 1 January and 31 December 2018 who had a diagnosis of T2DM, identified using ICD-10 version 2016 code E11.- for non-insulin dependent diabetes mellitus (NIDDM) [19], either as a primary or secondary diagnosis. A total of 6196 discharges who had code E11.- in the diagnosis records were identified. Discharges with complete information, including discharge disposition, were included in the final analysis. From the eligible T2DM-related discharges, a simple random sample of 2838 admission episodes was selected using IBM SPSS Statistics version 29.0 to obtain a representative and manageable analytic dataset for multivariable modeling.

### 2.3. Sample Size Calculation

A priori sample size was estimated for planning purposes using the Pocock formula, comparing two proportions. The expected proportions were derived from a study that reported in-hospital mortality rates for T2DM patients of 6.1% for surgical disciplines and 10.4% among medical disciplines [12]. Using a two-sided significance level of 0.05 and 80% statistical power, the calculated reference sample size was 2838.

From the eligible T2DM-related discharges, a simple random sample of 2838 admission episodes was selected using IBM SPSS Statistics version 29.0 to obtain a representative and manageable analytic dataset for multivariable modeling.

**Figure 1:**
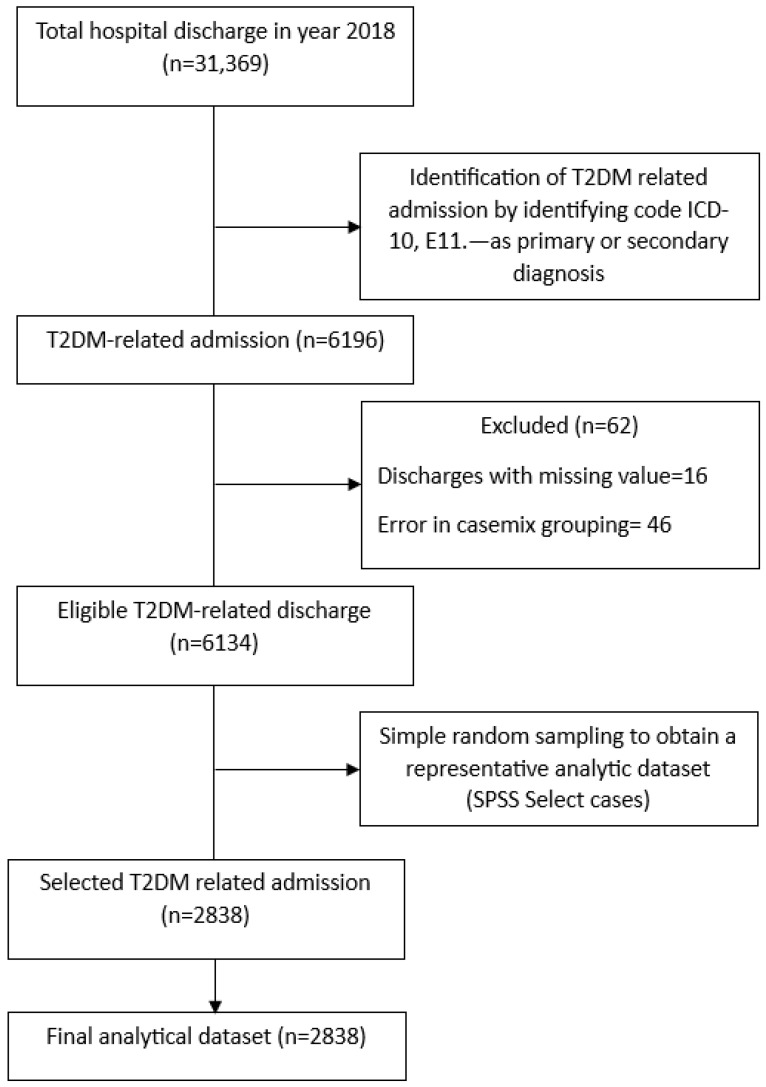
STROBE flowchart showing the selection of T2DM-related admission episodes included in the analysis.

A Strengthening the Reporting of Observational Studies in Epidemiology (STROBE)-compliant flow diagram (Figure 1) summarizes the selection process, including the number of discharges screened, excluded with reasons, and the final analytic sample of 2838 T2DM-related admission episodes included for analysis.

### 2.4. Variables

The dependent variable was in-hospital mortality (discharged dead vs. alive) while the independent variables that were investigated included demographic factors (gender, age (in years)) and clinical characteristics, including T2DM complications (further explained below); casemix main group (CMG), classified as shown in Table 1 (19); severity level (I, II and III); casemix base group (CBG) type (in-patient medical, in-patient surgical); hospital length of stay; and number of diagnosis coded for each patient. For analysis purposes, infrequent CMGs are classified as the “other” category.

T2DM complications were identified using the 4th edition of ICD-10 code E11.- and classified into four categories [20], as listed in Table 2. The data were anonymized and coded before analysis to ensure patient confidentiality.

### 2.5. Statistical Analysis

All statistical analyses were performed using IBM SPSS Statistics software, version 29.0 (IBM Corp., Armonk, NY, USA). Software details available at: https://www.ibm.com/products/spss-statistics, accessed on 20 January 2026. Descriptive statistics were used to summarize patient demographics and clinical characteristics. Categorical variables were presented as frequencies and percentages, while continuous variables were summarized using means and standard deviations.

Variables with a *p*-value < 0.25 in simple logistic regression were considered for inclusion in the multivariable logistic regression model. Multiple logistic regression was used to identify independent determinants of in-hospital mortality among T2DM-related admissions. As several clinically related variables were included to adjust for potential confounding, multicollinearity was assessed prior to model fitting using a variance inflation factor (VIF) and tolerance values to ensure the stability of regression estimates. No evidence of problematic multicollinearity was detected. Results are reported as adjusted odds ratios (aOR) with 95% confidence intervals (CI), and a *p*-value <0.05 was considered statistically significant.

Internal validation of the logistic regression model was conducted using nonparametric bootstrapping with 1000 resamples in IBM SPSS Statistics version 29. Bias-corrected accelerated (BCa) confidence intervals and bootstrap-adjusted standard errors were generated for all regression coefficients to evaluate stability and potential overfitting. Model discrimination was assessed using receiver operating characteristic (ROC) curve analysis, with the area under the curve (AUC), sensitivity, and specificity reported for the predicted probabilities of in-hospital mortality.

## 3. Results

### Descriptive Analysis

Table 3 shows the descriptive analysis of the study. A total of 2838 T2DM-related admissions were included in the study. The mean age of individuals involved in T2DM-related admissions during the study year was 65.22 years (SD 11.09) and gender distribution revealed a higher proportion of males, with 53.9% males compared to 46.1% females. With regard to complications, T2DM with no complications had the highest frequency of admissions, with 89.7%. and T2DM with acute complications had the lowest frequency of admissions, with 0.4%.

The in-hospital mortality proportion among T2DM-related admissions for the studied year was 4.2%. T2DM-related admissions resulting in in-hospital death involved individuals with a higher mean age (67.72 years, SD 12.06) compared to those who survived (65.11 (SD 11.03) years). Mortality was observed to be slightly higher in males (4.4%) compared to females (3.8%). Despite admissions without complications having the highest frequency among other complication categories, it had a lower mortality rate (4.2%) compared to acute complications (9.1%) and macrovascular complications (8.2%), while microvascular complications were associated with the least mortality (2.8%). No deaths were recorded among patients with unspecified complications.

Regarding CMG, admissions grouped under the infection and parasitic disease group had the highest mortality rate at 19.1%, followed by those in the respiratory system (6.8%), hepatobiliary and pancreatic system (5.5%), and central nervous system (5.0%). The lowest mortality rates were observed in the endocrine system (3.1%) and musculoskeletal/connective tissue disease (3.1%) groups.

For severity level, mortality increased with disease severity. Severity level 3 admissions exhibited a mortality rate of 7.7%, whereas severity level 2 and level 1 admissions showed lower rates of 1.8% and 1.6%, respectively. Length of stay was longer among admissions that resulted in mortality (8.7 (SD 8.4) days) compared to survivors (7.3 (SD 7.9) days). Similarly, the mean number of diagnoses was higher among T2DM-related admissions that resulted in mortality (8.8 (SD 3.1)) versus admissions who survived (7.0 (SD 2.7)).

Table 4 demonstrates the simple logistic regression and multiple logistic regression results of this study. Simple logistic regression identified several independent determinants of mortality among T2DM-related admissions, including age in years (OR = 1.022; 95% CI: 1.005, 1.040; *p* = 0.012) and CMG, including admissions with central nervous system conditions (OR = 3.517; 95% CI: 1.26, 9.817; *p* = 0.016), infections and parasitic diseases (OR = 15.859; 95% CI: 6.049, 41.58; *p* < 0.001), respiratory system diagnoses (OR = 4.873; 95% CI: 1.961, 12.112; *p* < 0.001), digestive system conditions (OR= 3.067, 95% CI: 1.1, 8.551; *p* < 0.032), and hepatobiliary and pancreatic system diseases (OR = 3.913; 95% CI: 1.236, 12.383; *p* = 0.02). A higher severity level was significantly associated with increased mortality among T2DM-related admissions. Severity level 3 showed a markedly increased risk compared to severity level 1 (OR = 5.021; 95% CI: 2.592, 9.728; *p* < 0.001) and number of diagnoses was also a significant determinant, with the odds of death increasing by 20.1% for each additional diagnosis (OR = 1.201; 95% CI: 1.139, 1.268; *p* < 0.001).

Several variables remained significant determinants of in-hospital mortality in the final model after adjusting for confounders. Infections and parasitic diseases remained a strong determinant of death (OR = 8.042; 95% CI: 2.999, 21.569; *p* < 0.001). Respiratory system diseases were also found to be independently associated with increased risk (aOR = 3.004; 95% CI: 1.192, 7.571; *p* = 0.020), similar to central nervous system (aOR = 3.484; 95% CI: 1.236, 9.826; *p* = 0.018) and hepatobiliary/pancreatic (aOR = 3.674; 95% CI: 1.143, 11.871; *p* = 0.029) conditions. These diagnostic categories formed part of the regrouped clinical casemix variable used in the bootstrap internal validation.

Severity level 3 was found to be an independent determinant of in-hospital mortality among T2DM-related admissions (aOR = 2.994; 95% CI: 1.464, 6.221; *p* = 0.003), confirming the stepwise increase in risk observed in the descriptive analysis. Number of diagnoses also showed a statistically significant association (aOR = 1.107; 95% CI: 1.032, 1.189; *p* = 0.005).

Conversely, age was not an independent determinant after adjusting for other variables (aOR = 1.009; 95% CI: 0.991, 1.027; *p* = 0.338). Length of stay was also found to be a significant determinant of mortality in the multivariate model (aOR = 0.989; 95% CI: 0.964, 1.014; *p* = 0.348). The model demonstrated good fit using the Hosmer–Lemeshow test (*p* = 0.156), with no significant multicollinearity (all VIFs < 10) and no influential outliers (Cook’s D < 1). The final multivariable model showed a Nagelkerke R^2^ value of 0.120, indicating a modest explanatory capacity of variability in in-hospital mortality. The model also showed acceptable predictive ability in sensitivity and specificity analyses.

Internal validation using 1000 bootstrap replications demonstrated that the multivariable logistic regression model was stable, with minimal evidence of overfitting. Bootstrap validation results are presented as log-odds coefficients (β) with bias-corrected and accelerated (BCa) 95% confidence intervals. The regrouped casemix category variable representing the diagnostic groups of infection-related, respiratory, hepatobiliary/pancreatic, and central nervous system, and other conditions retained significance after bootstrapping (β = 0.06; BCa 95% CI: 0.01–0.10), confirming the robustness of these group-level determinants. Severity level 2 (β = −0.31; BCa 95% CI: −1.94 to −0.58) and severity level 3 (β = −0.28; BCa 95% CI: −1.86 to −0.82) remained significant and robust determinants of in-hospital mortality among T2DM-related admissions. Number of diagnoses also showed consistent effects after bootstrapping (β = 0.10; BCa 95% CI: 0.04–0.15), indicating that multimorbidity is a stable determinant of mortality. Model performance was acceptable, with an AUC of 0.761 (95% CI: 0.720–0.802), demonstrating the reasonable ability of the model to discriminate between survivors and non-survivors.

## 4. Discussion

### 4.1. Statement of Principal Findings

In this study of determinants of in-hospital mortality among T2DM-related admissions in a Malaysian tertiary teaching hospital, we observed that in-hospital deaths among T2DM-related admissions occurred at a mean age of 67.72 years (SD 12.06).

Several key determinants of in-hospital mortality were identified to be significant. T2DM-related admissions with infections and parasitic diseases, central nervous system conditions, respiratory diseases, and hepatobiliary and pancreatic system conditions had significantly higher odds of dying in hospital compared to other admission diagnoses. These conditions are commonly associated with chronic hyperglycemia, which may impair immune defenses, healing, and physiological reserve [21].

Infections can rapidly decompensate diabetic patients, leading to multi-organ failure if not managed aggressively [22]. We also found that greater illness severity upon admission and a higher number of diagnoses or multimorbidity were strong independent determinants of in-hospital mortality among T2DM-related admissions. These results underscore the clinical reality that diabetic patients with complex and severe presentations represent a high-risk group in need of early identification and prompt, intensive management.

### 4.2. Interpretation Within the Context of the Wider Literature

Our results align with emerging evidence from recent studies in Southeast Asia and beyond. Notably, a large Indonesian analysis of over 610,000 hospitalized T2DM patients (2017–2022) reported an in-hospital mortality rate of 6.6%, with infections and severe complications as major contributors to death [12]. The mortality rate in the studied center showed a lower mortality rate of 4.2%, possibly reflecting differences in hospital level and case mix, as the Indonesian study included multiple tiers of hospitals (A, B, C, and D) according to the medical facilities. Meanwhile, this study was primarily conducted in a single tertiary teaching hospital in an urban area with advanced facilities and specialties. Additionally, another study in a single center examining in-hospital mortality in DM patients in Ethiopia showed an in-hospital mortality rate of 13.34%, which was 3 times higher than the mortality rate in this study [16], which may reflect differences in healthcare organization and resource availability. By contrast, a nationwide study in Poland reported that the mortality rate for T2DM patients was 3.3%, which was lower than in this study [17]. This difference may reflect advancements in the healthcare system in Poland in managing T2DM as a high-income economy country compared to Malaysia, which is an upper-middle-income economy.

#### 4.2.1. Infection-Related Determinants

Our finding that infection-related admissions carried higher mortality is also consistent with Indonesian data and other regional observations. For example, a study in Ethiopia reported that among diabetic inpatients who died, 37.5% had been admitted primarily due to infections [16]. A similar finding in a study in the United Kingdom investigating the contribution of infection to mortality in people with type 2 diabetes showed that infections were significantly associated with excess deaths among patients with T2DM.

#### 4.2.2. Respiratory System Diseases

Our study’s emphasis on respiratory disease as a mortality risk also echoes global data. A study in Poland also demonstrated that the presence of at least one disease of the respiratory system increased the odds of in-hospital mortality by five times (OR: 4.65; 95% CI: 4.03, 5.37; *p* < 0.001) [17]. Despite the data in the study being recorded before the COVID-19 pandemic, evidence from this study was witnessed during the pandemic, as it was found that T2DM was associated with significantly higher mortality in hospitalized COVID-19 patients. A study conducted on 7300 hospitalized patients with COVID-19 found that subjects with T2DM required more medical interventions and had a significantly higher mortality rate (7.8% compared to 2.7%) than non-diabetic individuals, with an adjusted hazard ratio (HR) of 1.49 [23].

#### 4.2.3. Hepatobiliary and Pancreatic Conditions

Non-alcoholic fatty liver disease (NAFLD) and type 2 T2DM often coexist [24]. This supports our finding that hepatobiliary/pancreatic conditions were associated with higher in-hospital mortality in the studied population. A study in China found that the risk of cardiovascular disease and overall death increased as NAFLD became more severe, especially in people with T2DM [25]. This finding was consistent with a study conducted in South Korea observing that patients with T2DM and without NAFLD had a higher five-year absolute risk for cardiovascular disease and all-cause death than those without T2DM and with grade 2 NAFLD [26].

#### 4.2.4. Central Nervous System (CNS) Involvement

A study performed in the United States investigating Parkinson’s Disease (PD) and T2DM mortality aligned with our study findings related to higher mortality among T2DM patients with central nervous system (CNS) conditions. Between 1999 and 2020, a total of 26,020 deaths occurred among older adults with T2DM and PD, suggesting that mortality among T2DM patients, particularly those with co-existing neurological conditions like Parkinson’s disease, may be heavily influenced by multiple demographic and clinical factors. These factors could contribute to the increased mortality observed in this population, with specific emphasis on how the progression of CNS disorders may exacerbate outcomes in T2DM patients [27].

#### 4.2.5. Multimorbidity and Number of Diagnoses

With regard to number of comorbidities, a prospective study in Ethiopia identified multiple comorbidities to be independent determinants of death, with patients having five or more comorbid conditions facing a 9–14-fold higher hazard of mortality [17]. This mirrors our result that a higher number of diagnoses reflecting comorbid illnesses markedly increased the mortality risk. This was also observed in Poland, where the presence of comorbidities was significantly related to prolonged length of stay and in-hospital mortality among patients with T2DM [17].

In contrast to some previous studies, age was not independently associated with in-hospital mortality after adjustment, likely reflecting confounding due to its strong correlation with multimorbidity and clinical severity. Age likely acts as a more distal factor, contributing to mortality risk through greater vulnerability, functional decline, comorbidity burden, and increased complexity of care. In line with recent evidence, advancing age remains an important driver of clinical deterioration and escalation of care among hospitalized adults [28].

When severity level and number of diagnoses were included in the multivariable model, these factors absorbed much of the risk typically attributed to age, resulting in a non-significant independent association. This finding differs from a study reporting that age increased the risk of death by 1.03 times per year (odds ratio 1.03, *p*-value < 0.05) [12]. This may be influenced by the advancement of care in the hospital in our study as a tertiary teaching hospital in Kuala Lumpur compared to the study conducted involving various levels of hospital care in the mentioned study, where the median age was 57 years old compared to 65.22 years in our study [29]. Despite investigating other points of view with regard to age, various studies found that earlier age of T2DM diagnosis is a determinant of mortality among T2DM patients [30].

### 4.3. Study Limitation and Strength

This study has several limitations that require consideration. Firstly, our dataset does not allow complete identification of unique patients across hospitalizations as the data were anonymized to ensure patient confidentiality. Therefore the analysis conducted was at the discharge (admission) level rather than individual patient level. Due to the analysis being conducted at the admission level, a single individual may have contributed more than one hospitalization. Therefore, results should not be interpreted as patient-level mortality risk.

Despite identified associations between determinant factors and in-hospital mortality, the retrospective cross-sectional design limits the ability to infer causal relationships between the investigated factors and in-hospital mortality among T2DM admissions. The study utilized secondary data extracted from the hospital’s casemix database, which were systematically coded. The data may be subject to coding inaccuracies contributed by incomplete clinical documentation. The accuracy of diagnosis and severity level classifications are highly dependent on the quality of these two mentioned factors, as the process of clinical documentation and clinical coding is still performed manually in this center. Additionally, the number of diagnoses captured may not fully reflect the patient’s comorbidity burden due to possible under-documentation or misclassification, potentially underestimating the complexity of cases.

The next important limitation is that several potentially clinical parameters were not included in the analysis, as they were not available in the database. These include biochemical indicators such as patient HbA1c level, glycemic control, and treatment regimens prescribed to the patient. Socioeconomic factors, such as income level and education, that may influence clinical outcomes were also not investigated. The analysis utilized data from 2018 from a single tertiary hospital in an urban setting. Generalization may be limited to the general population and other healthcare settings; however, the study provides a good picture of T2DM in a healthcare setting, especially in a tertiary hospital that serves as a referral center. Changes in clinical practice guidelines, hospital policies, or patient demographics may affect the current applicability of the results.

Despite its limitations, this study has several notable strengths. First, it utilizes a large and systematically collected hospital-wide casemix dataset, enabling the analysis of a large number of T2DM-related admissions over a full calendar year. Second, the use of a nationally standardized casemix system allows for an understanding of the relationships between disease complexity, resource utilization, and in-hospital mortality among T2DM-related admissions.

### 4.4. Implications for Policy, Practice, and Research

The study is among the few to investigate in-hospital mortality determinants among T2DM-related admissions in Malaysia using real-world hospital data from a tertiary teaching institution. As such, the findings contribute to evidence that can inform both clinical management strategies and health system policy-making, particularly in similar middle-income country settings.

The findings from this study highlight the opportunity for targeted clinical risk stratification among T2DM-related admissions. T2DM-related admissions with infections, respiratory illnesses, hepatobiliary and pancreatic conditions, CNS conditions, and multiple comorbidities face significantly higher mortality risk and should be prioritized for diligent monitoring and multidisciplinary care. Identifying these high-risk groups at the point of admission provides clinicians with a clear opportunity to prioritize closer monitoring, earlier escalation of care, and multidisciplinary involvement. Hospitals could implement early warning systems tailored to diabetic patients, consider intensive care unit (ICU) consults at earlier stages, and vigorous glycemic control, infection management, and rehabilitation support. Hospital managers, together with clinical specialists, should work together on strategy development to address the identified issues and implement solutions.

From a health system perspective, this study demonstrates the utility of casemix administrative data as a practical tool for routine risk profiling and performance monitoring. Hospital managers and clinical leaders can leverage these findings to improve resource allocation, strengthen clinical pathways, and support quality improvement initiatives aimed at reducing preventable in-hospital deaths. Incorporating admission-level risk factors into hospital dashboards or mortality review frameworks can help institutions identify vulnerable patient groups more accurately and intervene earlier.

From a public health perspective, the high mortality rates observed among severe T2DM-related admissions highlight gaps in outpatient and primary care management. This portrays patients arriving at hospitals late in the disease process. Strengthening community-based care through diabetes education, home monitoring by trained nurses, and early identification of alarming conditions by patients can assist rapid referral systems from primary to tertiary care. This will help detect and manage complications earlier. Eventually, improving outcomes will require a dual approach: preventing severe illness through better chronic disease management and optimizing inpatient care for those who do become critically ill. Each identified risk factor offers a pathway for targeted intervention, helping to reduce T2DM-related in-hospital deaths and lessen the growing burden on tertiary healthcare services.

## 5. Conclusions

This study identified key determinants of in-hospital mortality among T2DM-related admissions in a tertiary teaching hospital, which included infections, respiratory diseases, hepatobiliary and pancreatic conditions, central nervous system conditions, and multiple comorbidities. These findings highlight the necessity for early stratification, intensive clinical management, targeted training for frontline teams managing acute metabolic and infectious complications, and a multidisciplinary approach for high-risk groups. Hospital management should expand intensive care capacity to better manage complex cases, specifically T2DM in this context, and may benefit from strengthening clinical pathways for severe T2DM cases.

At a broader level, the findings point to opportunities for the Malaysian healthcare system to enhance hospital management of T2DM through better integration of casemix data in clinical decision-making, improved coordination between primary and tertiary care, and strategic resource allocation for high-risk patient groups. Strengthening these system-level processes can support more responsive, equitable, and effective care, ultimately helping to reduce preventable in-hospital deaths among T2DM-related admissions.

## Figures and Tables

**Table 1:** Case Main Group in Casemix System Using ICD-10.

Description	CMG Code
Central nervous system group	G
Respiratory system group	J
Cardiovascular system group	I
Digestive system group	K
Hepatobiliary and pancreatic systems group	B
Musculoskeletal system and connective tissue group	M
Skin, subcutaneous tissue, and breast group	L
Endocrine system, nutrition, and metabolism group	E
Nephro-urinary system group	N
Infectious and parasitic diseases group	A
Hematopoeitic and immune system group	D
Factors influencing health status and other contacts with health services group	Z
Eye and adnexa group	H
Myeloproliferative system and neoplasm group	C
Injuries, poisoning, and toxic effects of drugs group	S
Female reproductive system group	W
Ear, nose, mouth, and throat group	U
Male reproductive system group	V
Mental health and behavioral group Substance abuse and dependence	F
delivery group	O

**Table 2:** Classification of T2DM complications according to 4th edition of ICD-10.

ICD-10 Code	ICD-10 Description	Complication Classification
E11.0	T2DM with coma	Acute complications
E11.1	T2DM with ketoacidosis	Acute complications
E11.2	T2DM with renal complications	Microvascular complications
E11.3	T2DM with ophthalmic complications	Microvascular complications
E11.4	T2DM with neurological complications	Microvascular complications
E11.5	T2DM with peripheral circulatory complications	Macrovascular complications
E11.6	T2DM with other specified complications	Unspecified complications
E11.7	T2DM with multiple complications	Unspecified complications
E11.8	T2DM with unspecified complications	Unspecified complications
E11.9	T2DM with no complications	Without complications

T2DM: Type 2 diabetes mellitus; ICD-10: International Classification of Diseases, 10th Revision.

**Table 3:** Descriptive analysis of T2DM-related admissions (N = 2838).

	All T2DM-Related Admissions		Type 2 Diabetes Discharge Status			
			Alive		Death	
	n (%)	Mean (SD)	n (%)	Mean (SD)	n (%)	Mean (SD)
In-hospital mortality proportion			2720 (95.8)		118 (4.2)	
Age in year		65.22 (11.09)		65.11 (11.03)		67.72 (12.06)
Gender						
Male	1530 (53.9)		1462 (95.6)		68 (4.4)	
Female	1308 (46.1)		1258 (96.2)		50 (3.8)	
T2DM complications						
No complications	2547 (89.7)		2440 (95.8)		107 (4.2)	
Macrovascular	73 (2.6)		67 (91.8)		6 (8.2)	
Microvascular	141 (5.0)		137 (97.2)		4 (2.8)	
Acute	11 (0.4)		10 (90.9)		1 (9.1)	
Unspecified	66 (2.3)		66 (100)		0 (0)	
Casemix base group (CBG) type						
Inpatient medical	2234 (78.7)		2139 (95.6)		95 (4.3)	
Inpatient surgical	604 (21.3)		581 (96.2)		23 (3.8)	
Casemix main group (CMG)						
Infection and parasitic diseases	89 (3.1)		72 (80.9)		17 (19.1)	
Nephro-urinary system	311 (11.0)		303 (97.4)		8 (2.6)	
Endocrine system, nutrition, and metabolism	162 (5.7)		157 (96.9)		5 (3.1)	
Skin, subcutaneous tissue, and breast	142 (5.0)		137 (96.5)		5 (3.5)	
Musculoskeletal system and connective tissue	223 (7.9)		216 (96.9)		7 (3.1)	
Hepatobiliary and pancreatic system	109 (3.8)		103 (94.5)		6 (5.5)	
Digestive system	229 (8.1)		219 (95.6)		10 (4.4)	
Cardiovascular system	622 (21.9)		601 (96.6)		21 (3.4)	
Respiratory system	340 (12.0)		317 (93.2)		23 (6.8)	
Central nervous system	201 (7.1)		191 (95.0)		10 (5.0)	
Other *	410 (14.4)		403 (98.5)		7 (1.5)	
Severity level						
Level 1	612 (21.6)		602 (98.4)		10 (1.6)	
Level 2	1070 (37.7)		1051 (98.2)		19 (1.8)	
Level 3	1156 (40.7)		1067 (92.3)		89 (7.7)	
Length of stay (days)		7.4 (7.9)	7.3 (7.9)			8.7 (8.4)
Total number of diagnoses		6.2 (2.8)	7.0 (2.7)			8.8 (3.1)

* CMG classified into other groups includes: hematopoeitic and immune system group; factors influencing health status and other contacts with health services group; eye and adnexa group; myeloproliferative system and neoplasm group; injuries, poisoning and toxic effects of drugs group; female reproductive system group; ear, nose, mouth, and throat group; male reproductive system group; mental health and behavioral group; substance abuse and dependence delivery group.

**Table 4:** Simple and multiple logistic regression analyses of determinants of in-hospital mortality among T2DM-related admissions.

	Simple Logistic Regression				Multiple Logistic Regression			
	Crude OR	(95% CI)		*p*-Value	aOR	(95% CI)		*p*-Value
Sex								
Female	1 (reference)							
Male	1.17	0.806	1.699	0.409				
Age (year)	1.022	1.005	1.04	0.012 *	1.009	0.991	1.027	0.338
T2DM complications				0.408				
No complications	1 (reference)							
Macrovascular	0.103	2.042	0.866	4.813				
Microvascular	0.431	0.666	0.242	1.833				
Acute	0.434	2.28	0.289	17.976				
Unspecified	0	0		0.999				
Casemix main group				<0.001 *				0.002
Other ⁱ	1 (reference)				1 (reference)			
Central nervous system	3.517	1.26	9.817	0.016 *	3.484	1.236	9.826	0.018
Respiratory system	4.873	1.961	12.112	<0.001 *	3.004	1.192	7.571	0.020
Cardiovascular system	2.347	0.939	5.866	0.068	1.941	0.771	4.889	0.159
Digestive system	3.067	1.1	8.551	0.032 **	2.764	0.981	7.785	0.054
Hepatobiliary and pancreatic system	3.913	1.236	12.383	0.020 *	3.674	1.143	11.81	0.029
Musculoskeletal system and connective tissue	2.177	0.722	6.558	0.167 **	2.549	0.833	7.799	0.101
Skin, subcutaneous tissue, and breast	2.451	0.736	8.159	0.144 **	2.367	0.702	7.981	0.165
Endocrine system, nutrition, and metabolism	2.139	0.644	7.109	0.215 **	1.593	0.473	5.363	0.452
Infection and parasitic diseases	15.859	6.049	41.58	<0.001 *	8.042	2.999	21.569	<0.001
Severity level				<0.001 *				<0.001
Severity level 1	1 (reference)				1 (reference)			
Severity level 2	1.088	0.503	2.356	0.830	0.914	0.416	2.008	0.823
Severity level 3	5.021	2.592	9.728	<0.001 *	2.994	1.441	6.221	0.003
CBG type								
Inpatient surgical	1 (reference)							
Inpatient medical	1.122	0.705	1.785	0.627				
Length of stay	1.018	1 (reference)	1.036	0.057 **	0.989	0.964	1.014	0.380
Total number of diagnoses	1.201	1.139	1.268	<0.001 *	1.107	1.032	1.189	0.005

CI = confidence interval, OR = odds ratio, aOR = adjusted OR, T2DM = type 2 diabetes mellitus. 1 as reference. Factors with * *p* < 0.05 and ** *p* < 0.25 were included in the multiple logistic regression (MLR) analysis. Method for MLR: Forward LR with Nagelkerke R2: 0.120. Notes: 1. Variance inflation factor (VIF) for all selected variables is less than 10; 2. Hosmer–Lemeshow goodness of fit test shows model fits well (*p* = 0.156); 3. The model demonstrates good predictive ability with sensitivity and specificity values reported: 95.8% of cases are predicted correctly; 4. No influential outliers were detected (Cook’s influential statistic is less than 1.0). ⁱ CMG classified in other groups include hematopoeitic and immune system group; factors influencing health status and other contacts with health services group; eye and adnexa group; myeloproliferative system and neoplasm group; injuries, poisoning, and toxic effects of drugs group; female reproductive system group; ear, nose, mouth, and throat group; male reproductive system group; mental health and behavioral group; substance abuse and dependence delivery group.

## Data Availability

The data that support the findings of this study are available upon request from the corresponding author due to privacy and ethical restrictions.

## Abbreviations

The following abbreviations are used in this manuscript:

T2DM	Type 2 diabetes mellitus
HCTM	Hospital Canselor Tuanku Muhriz
NCD	Non-communicable disease
IDF	International Diabetes Federation
NHMS	National Health and Morbidity Survey
NDR	National Diabetes Registry
UKM	University Kebangsaan Malaysia
ICD-10	International Classification of Diseases, 10th Revision
ICD-9-CM	ICD-9 Clinical Modification
MY-DRG^®^	Malaysian Diagnosis-Related Group
CMG	Casemix main group
NIDDM	Non-insulin dependent diabetes mellitus
CBG	Casemix base group
NAFLD	Non-alcoholic fatty liver disease
PD	Parkinson’s Disease
CNS	Central nervous system
